# Muscle Architectural and Functional Adaptations Following 12-Weeks of Stretching in Adolescent Female Athletes

**DOI:** 10.3389/fphys.2021.701338

**Published:** 2021-07-16

**Authors:** Ioli Panidi, Gregory C. Bogdanis, Gerasimos Terzis, Anastasia Donti, Andreas Konrad, Vasiliki Gaspari, Olyvia Donti

**Affiliations:** ^1^Sports Performance Laboratory, School of Physical Education and Sport Science, National and Kapodistrian, University of Athens, Athens, Greece; ^2^Institute of Human Movement Science, Sport and Health, University of Graz, Graz, Austria

**Keywords:** flexibility, muscle fascicle length, muscle cross-sectional area, puberty, triceps surae

## Abstract

This study examined the effects of high-volume static stretching training on gastrocnemius muscle architecture, ankle angle and jump height in 21 female adolescent volleyball players. Static stretching of the plantar flexors of one leg (STR) was performed five times/week for 12 weeks, in addition to volleyball training, with the contra-lateral leg used as control (CON). Total duration of stretching per session increased from 540 s (week 1) to 900 s (week 12). At baseline, week 12 and after 3 weeks of detraining, muscle architecture at the middle and the distal part of both gastrocnemius heads (medialis and lateralis) and ankle angle were examined at rest and at maximum dorsiflexion. At the same time-points gastrocnemius cross-sectional area (CSA) was also assessed, while jumping height was measured at baseline and week 12. Following intervention, ankle dorsiflexion increased in both legs with a greater increase in STR than CON (22 ± 20% vs. 8 ± 17%, *p* < 0.001). Fascicle length at the middle part of gastrocnemius medialis increased only in the STR, at rest (6 ± 7%, *p* = 0.006) and at maximum dorsiflexion (11 ± 7%, *p* < 0.001). Fascicle length at maximum dorsiflexion also increased at the distal part of gastrocnemius lateralis of STR (15 ± 13%, *p* < 0.001). A greater increase in CSA (23 ± 14% vs. 13 ± 14%, *p* < 0.001) and in one-leg jumping height (27 ± 30% vs. 17 ± 23%, *p* < 0.001) was found in STR than CON. Changes in ankle angle, fascicle length and CSA were maintained following detraining. High-volume stretching training for 12 weeks results in ankle dorsiflexion, fascicle length and muscle cross sectional area increases in adolescent female volleyball players. These adaptations may partly explain improvements in jump performance.

## Introduction

Skeletal muscle tissue remodels its structure in response to mechanical loading, growth (Bénard et al., 2011) and type of muscle contraction (Franchi et al., 2017). Although muscle plasticity is limited by cell lineage during growth, mechanical loading triggers molecular and structural changes that reprogram metabolism and may modify the physiological (Ferraro et al., 2014) and contractile properties of muscle fibers (Franchi et al., 2014).

Mechanical loading may be applied on the muscles in different forms (e.g., concentric or eccentric contraction), that induce distinct adaptations in muscle architecture (Franchi et al., 2017). Muscle stretching may also be considered as a form of mechanical loading, but to date, there is limited and conflicting evidence regarding its effects on architectural parameters (Andrade et al., 2020; Moltubakk et al., 2021; Yahata et al., 2021). Some studies found significant increases in fascicle length (Freitas and Mil-Homens, 2015; Simpson et al., 2017; Andrade et al., 2020) following 6–12 weeks of stretching training, while there is evidence that morphological changes are heterogeneous across muscle length or between muscles (Simpson et al., 2017). Fascicular length increases were associated with trivial changes in fascicle pennation angles and thickness (Simpson et al., 2017). Moreover, Nakamura et al. (2012) and Blazevich et al. (2014), reported increased muscle extensibility after 3 and 4 weeks of static stretching training, respectively. In contrast, other studies failed to detect changes in muscle architecture following stretching interventions (Blazevich et al., 2014; Konrad and Tilp, 2014). As Freitas et al. (2018) suggested, discrepant results between studies may be due to the different stretching protocols applied. For example, longer-term stretching interventions (Andrade et al., 2020), overloaded static stretching (Simpson et al., 2017) and/or high-intensity and long-duration stretching bouts (Freitas and Mil-Homens, 2015) were more effective in triggering morphological adaptations in the muscle-tendon unit.

Muscle architecture is also affected by maturational growth, as muscle size and length adapt to meet the functional demands of daily life (Weide et al., 2015). Previous cross-sectional studies reported that from childhood to adolescence both, the length component of the physiological cross-sectional area (CSA) of gastrocnemius medialis and, fascicle length increase, proportionally to the increased tibia length, indicating growth of muscle fibers and fascicles in length and in diameter, with the latter contributing most (Bénard et al., 2011). In contrast, no changes were reported in gastrocnemius medialis pennation angles during growth (O’Brien et al., 2010; Bénard et al., 2011) and this implies that the increase in fascicle diameters within muscle is accommodated by a simultaneous and proportional increase in aponeurosis length (Binzoni et al., 2001). Since both growth and mechanical load affect muscle architecture, it would be interesting to study the effects of stretching interventions on muscle architecture and function in growing children and adolescents, which are largely unknown. This would also have a significant practical impact, since in some sports (e.g., gymnastics, dance), growing athletes are submitted to frequent and excessive flexibility training (Donti et al., 2018).

Changes in muscle morphology following stretching training are thought to be related to joint range of motion (ROM), which, in turn, is an important component of physical fitness and health (Magnusson and Renström, 2006). Enhanced ROM may increase the distance over which muscle force is applied or absorbed and allows athletes to demonstrate strength through the ROM typically used in their sport. Acute or short-term (2–8 weeks) increases in joint ROM following stretching are usually attributed to increased stretch tolerance and/or are related to a decreased joint resistance to stretch (Magnusson, 1998). However, decreased passive torque levels (Guissard and Duchateau, 2004) despite increased ROM indicate that the repeated stretching stimulus may modify the stiffness of the musculotendinous unit (Nakamura et al., 2020). Thus, stretching interventions of longer duration and intensity are required to examine the effect of stretching on muscle morphological characteristics and ROM. Larger ROM following training is associated with increased muscle contractile function (Ferreira et al., 2007; Moltubakk et al., 2016) and greater jump height (Kokkonen et al., 2007), possibly through the addition of sarcomeres in series that enables higher contraction velocity and a more optimal sarcomere length across a wider range of joint angles (Lieber and Fridén, 2000). Interestingly, from infancy to adulthood, ankle joint ROM decreases about 1.5% per year (Bénard et al., 2011), and during adolescence levels of flexibility tend to plateau or decrease at the time of the adolescent spurt (Malina, 2007).

Collectively, evidence regarding muscle morphological changes following stretching training in adolescents, as well as the optimal loading characteristics of the stretching protocols is inconclusive (Freitas et al., 2018). Moreover, no study has examined changes in muscle architecture following high-volume stretching training in developing athletes and whether these changes are homogeneous between muscles or across muscle length of a bipennate muscle, such as gastrocnemius. Last, it is not known if ROM increases following stretching training are associated with changes in jump performance. Thus, the aim of this study was to examine the effects of 12 weeks of static stretching training and 3 weeks of detraining on functional and architectural parameters in adolescent female volleyball athletes. It was hypothesized that ankle dorsiflexion would increase following intervention. Fascicle length at rest and at maximum dorsiflexion stretching was expected to increase while muscle thickness, anatomical CSA and pennation angle were hypothesized to remain unchanged. Jump performance was also expected to increase.

## Materials and Methods

### Participants

An *a priori* sample size calculation (primary outcome fascicle length) for a repeated measures ANOVA based on the literature (Freitas and Mil-Homens, 2015) suggested a group size of at least 16 participants to obtain a medium effect (effect size f: 0.335, alpha = 0.05, power = 0.8, correlation among measures 0.5). Twenty-six female volleyball players, aged 13–15 years, were initially recruited for the study. All participants were from the same volleyball club and trained with the same coach in two groups of 12–14 athletes each. Athletes performed 6 h of volleyball training weekly (4/week), without additional strength or endurance training and participated in competitions according to the national age-category calendar. Each volleyball training session included technical and tactical skills. An experienced coach recorded all the skills with a plyometric component for the lower limbs (e.g., block and spike drills), that were performed during training sessions to ensure that all the athletes received similar training load. The characteristics of the participants are shown in Table 1. Exclusion criteria were lower limb injury over the past 6 months, missing more than three training sessions during intervention, and previous history of systematic stretching training. Four athletes missed more than three training sessions and one had an ankle sprain, not associated with the intervention, and were excluded from the study. In total, 21 adolescent volleyball players completed the study. No stretching-associated injury was reported during the study.

**Table 1:** Characteristics (mean ± SD) of the participants.

	Baseline (*n* = 21)	Week 12 (*n* = 21)	Cohen’s *d*	*p*
Age (years)	13.5 ± 1.4	13.7 ± 1.4	0.15	0.257
Height (cm)	159.8 ± 7.0	160.1 ± 6.9	0.03	0.004
Sitting height (cm)	85.4 ± 3.5	85.4 ± 3.5	0.00	0.176
Calf length (cm)	37.2 ± 2.2	37.2 ± 2.2	0.44	0.666
Body mass (kg)	53.7 ± 7.6	53.6 ± 7.7	0.01	0.909
BMI (kg/m^2^)	21.0 ± 2.8	20.9 ± 2.8	0.03	0.609
Maturity offset	−0.8 ± 0.9	−0.6 ± 0.9	0.19	0.001

Maturity offset was calculated at baseline and week 12, according to the prediction equation of Mirwald et al. (2002). In addition, athletes recorded their menstrual cycle in a personal diary throughout the study. Ten of the twenty-one athletes were prior to menarche. Anthropometric and maturity characteristics of the participants are shown in Table 1. Prior to the start of the study, participants and their parents were informed about the purpose and risks involved, the testing and training procedures, and their right to terminate participation at will and gave their informed consent. The study was approved by the Institutional Ethics Committee (registration number: 1040, 14/02/2018) in accordance with the World Medical Association’s Declaration of Helsinki.

### Experimental Design

A 15-week within-subjects controlled design was used in this study. The athletes performed for 12 weeks supplementary to their typical volleyball training, static stretching of the plantar flexors, of one leg (stretched leg; STR) while the contra-lateral leg served as control (control leg; CON). The assignment of the legs was done in a random and counterbalanced order, so that half of the participants stretched their dominant leg (leg preferred to kick a ball) while the other half stretched their non-dominant leg. Furthermore, assignment of the legs was such that the STR and CON legs were similar in resting fascicle lengths and CSA of gastrocnemius medialis and lateralis, ankle angle at rest and during maximum dorsiflexion, and jumping height (Table 2). The testing order of the legs was randomized at the first session and repeated identically at subsequent sessions. All measurements were undertaken by the same investigators who were blinded to the leg assignment.

**Table 2:** Baseline evaluation of the gastrocnemius heads fascicle length and muscle anatomical cross-sectional area (CSA), ankle angle and jump performance in 21 adolescent volleyball players used for the legs’ assignment.

	Control leg (*n* = 21)	Stretched leg (*n* = 21)	Cohen’s *d*	*p*
Fascicle length, Medialis, MP (cm)	4.7 ± 0.7	4.8 ± 0.6	0.10	0.589
Fascicle length, Medialis, DP (cm)	4.5 ± 0.7	4.5 ± 0.6	0.05	0.785
Fascicle length, Lateralis, MP (cm)	4.7 ± 0.6	4.8 ± 0.8	0.09	0.574
Fascicle length, Lateralis, DP (cm)	4.4 ± 0.6	4.6 ± 0.8	0.28	0.184
One-CMJ height (cm)	6.3 ± 1.8	6.5 ± 1.8	0.13	0.239
Ankle angle at rest (^0^)	113.3 ± 6.2	113.2 ± 5.9	0.03	0.763
Ankle angle during stretching (^0^)	62.8 ± 3.7	62.8 ± 3.4	0.00	0.995
Anatomical CSA (cm^2^)	16.1 ± 3.8	16.3 ± 3.4	0.06	0.611

*MP, middle part of the muscle; DP, distal part of the muscle*.

**Figure 1:**
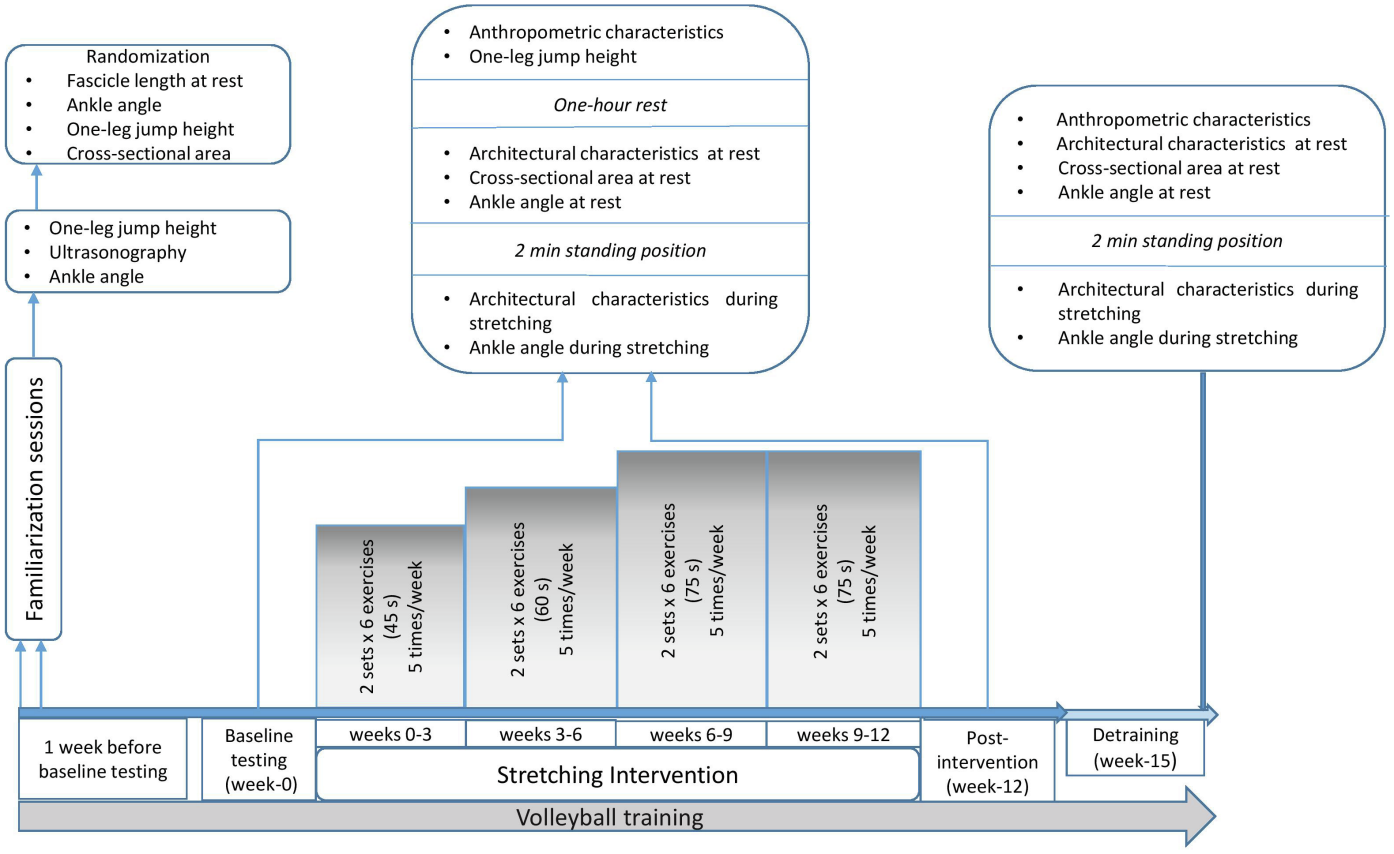
Schematic representation of the experimental design.

Participants visited the lab four times. Two familiarization sessions were performed 1 week before the intervention, where participants underwent ultrasound assessment of the gastrocnemius medialis and lateralis and were familiarized with the stretching training and testing procedures. In addition, intra-class correlation coefficient (ICC) was calculated for all the examined parameters and data were used for the randomization process. Following familiarization, a pre-intervention testing session (week 0), a post-intervention (week 12) -held 48 h after the last stretching training- and a detraining testing session (week 15) were performed (Figure 1). At weeks 0, 12 and 15, ultrasonography was applied on both legs, to measure fascicle length, pennation angle, and muscle thickness at the middle (MP) and the distal part (DP) of gastrocnemius medialis and lateralis, at rest and at maximum dorsiflexion. At the same time points (weeks 0, 12 and 15), ankle angle was measured at rest and at maximum dorsiflexion for both legs, as well as gastrocnemius heads (medialis and lateralis) anatomical CSA. One-leg countermovement jump (CMJ) height for the STR and the CON was assessed pre- and post-intervention (weeks 0 and 12, respectively; Figure 1). Due to COVID-19 restrictions, no evaluation of CMJ in week 15 was performed. Ultrasonography and ankle angle measurements at week 15, were performed in the athletes training facilities. Athletes refrained from stretching and training 48 h prior to testing sessions. During the study, all athletes maintained their habitual diet, and did not take any medications or nutritional supplements.

### Assessments

#### Anthropometric Characteristics

Anthropometric characteristics of the athletes were assessed at the beginning of every testing session, to monitor maturation and growth (Figure 1). Height was measured using a stadiometer (Seca 208, Hamburg, Germany), sitting-height was measured with a hand-made scale on the wall, and body mass was measured with a calibrated digital scale (Seca 710, Hamburg, Germany). Leg length was calculated as the distance between trochanter major to the floor while standing. Calf length was measured as the distance between tibiofemoral joint to the most prominent point of medial malleolus. Body mass index was calculated as the ratio of body mass to the squared standing height (kg/m^2^). All measurement sessions were performed in the morning hours at room temperature of 21–23°C.

#### Countermovement Jump Height

One hour before ultrasound measurements, the participants performed one-leg CMJ with the STR and CON legs, in a random and counterbalance order (Figure 1). Jumps were recorded using a force platform (Applied Measurements Ltd. Co. United Kingdom, WP800) interfaced with a computer (software Kyowa sensor interface PCD-320A, Kyowa Electronic Instruments Co., Ltd., Japan, sampling frequency 1 kHz). Each athlete started from a balanced position with the arms akimbo and after a preliminary downward movement, they immediately performed a vertical jump with the ankle and knee fully extended and landed with the same body configuration. Athletes were instructed to perform two maximal efforts with 2 min rest between jumps. The force-time curve of the highest jump of each leg was used for further analysis. Jumping height was calculated based on take-off velocity, according to the following equation: jumping height = (take-off-velocity)^2^/2 g. ICC for one-leg CMJ was 0.899 (95% CI: 0.516–0.948, CV = 16%, SEM = 5%).

#### Ultrasonography Assessment

Gastrocnemius muscle architecture was evaluated using a 10 MHz linear probe (38 mm) *via* extended field-of-view mode (Product model Z5, Shenzhen, Mindray Bio-Medical Electronics Co., Ltd., Shenzhen, China), in two conditions: (1) at rest, with the participants lying in prone position with their foot hanging freely off a physiotherapy bed, and (2) during wall calf stretching (Jung et al., 2009). During wall calf stretching, the athletes were instructed to move slowly against the wall for 45 s until they reached the point of discomfort. For the last 15 s of the 1-min calf wall stretching, the athletes remained still. Ultrasonography for the one gastrocnemius head (medialis or lateralis in a random order) started approximately 10 s before the end of the 1 min stretching and the other head followed. Ultrasonography was finished approximately 10 s later. If an image was not clear, the measurement was repeated after 1 h of rest to avoid the conditioning effect of the repeated stretching.

**Figure 2:**
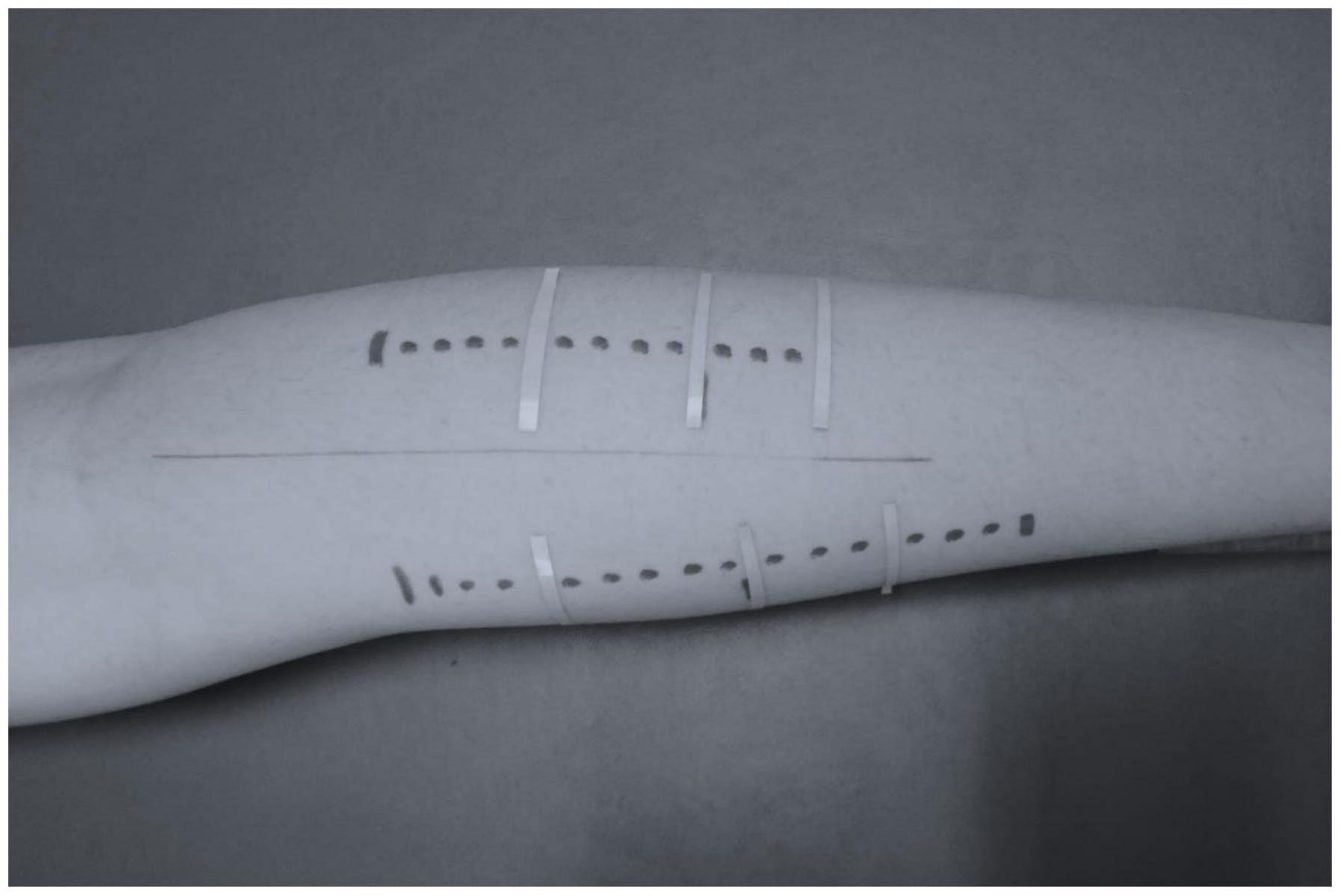
Probe path for ultrasonography assessment of gastrocnemius medialis and lateralis.

Panoramic ultrasound images were obtained to avoid trigonometric estimations or multiple scans along the muscle, and to capture fascicles longer than the probe’s length. The athletes remained in prone position for at least 20 min, while anatomical markers were placed in both legs. Echo-absorptive markers were placed at the one-third (30%) and at the half (50%) of the distance between popliteal crease to the center of medial malleolus to distinguish the MP and the DP of gastrocnemius medialis and lateralis, respectively by using real-time static ultrasound imaging. An echo-absorptive tape was placed at the musculotendinous junction (MTJ), as well. The probe was orientated parallel to the fascicles and by real-time imaging, a probe-path was drawn on the skin with a permanent marker in order to obtain the same field of view in each measurement (Panidi et al., 2020; Figure 2).

**Figure 3:**
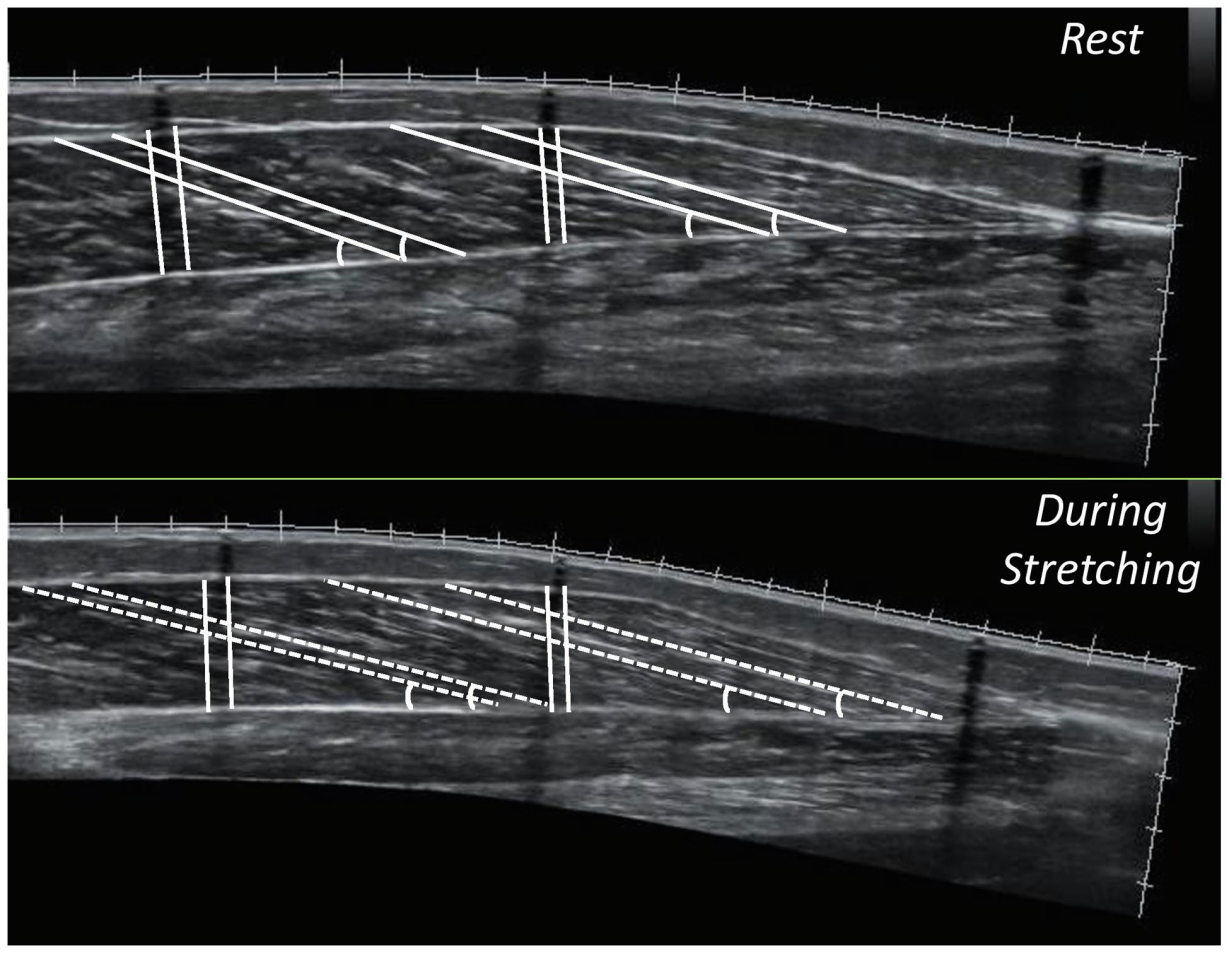
Panoramic sonographic images of gastrocnemius medialis showing fascicle length, pennation angle and muscle thickness, at the middle (MP) and the distal part (DP) of the muscle of the same athlete (top panel: at rest, bottom panel: during stretching).

Aquasonic gel was used to provide acoustic contact between the skin and the probe. The path started 38 mm above the middle skin marker and the probe was moved steadily and slowly along the drawn path. A panoramic view was taken for each gastrocnemius head (medial and lateral) until the point where the probe was crossing the MTJ marker. For each part (middle and distal) of the gastrocnemius medialis and lateralis, two different fascicles and their respective pennation angles were measured, in both legs. In addition, two consecutive measurements of muscle thicknesses were performed. For further analysis, the average values were used (Figure 3).

**Figure 4:**
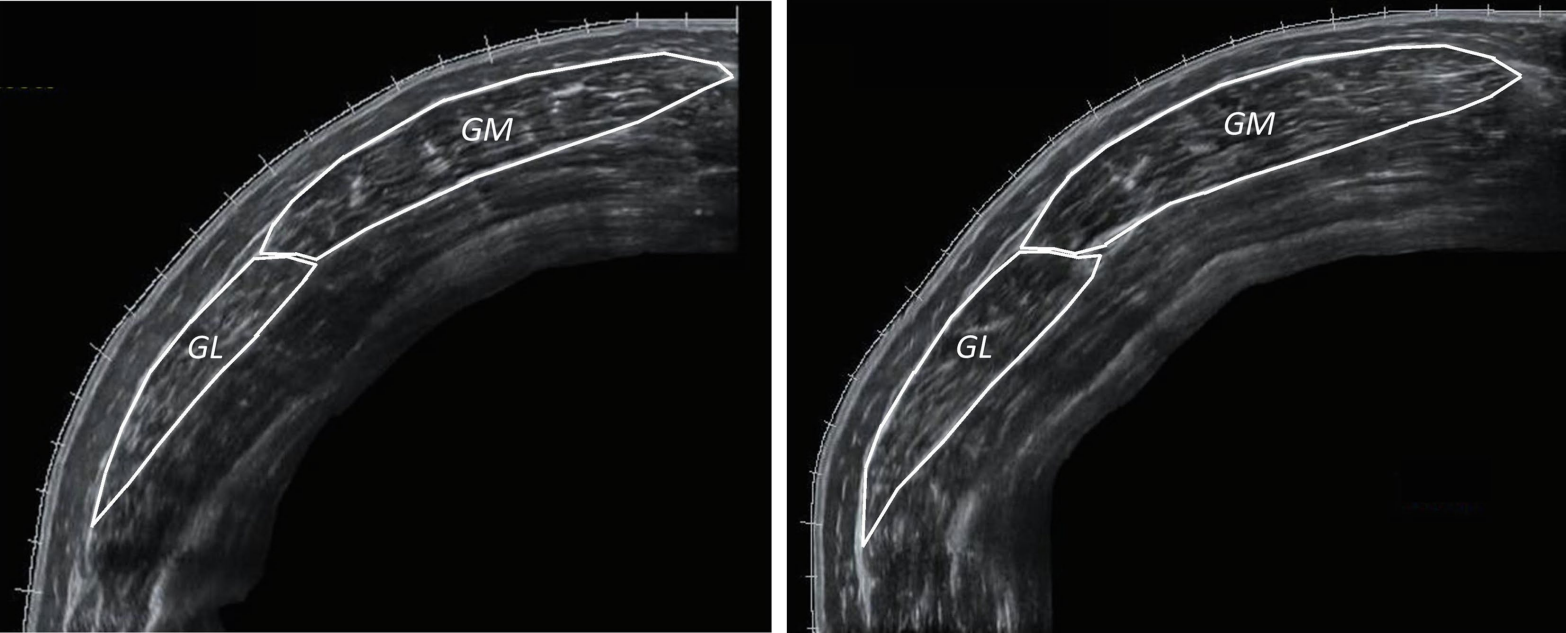
Anatomical CSA of the stretched leg (STR) of the same participant’s gastrocnemius medialis (GM) and lateralis (GL; left: week-0, right: week-12).

Gastrocnemius (medialis and lateralis) CSA was measured at rest, at the MP of the muscle, with extended field-of-view mode. By using static real time ultrasonography, an axial perpendicular line was drawn from the origin of gastrocnemius medialis to the respective origin of gastrocnemius lateralis, at the MP of gastrocnemius muscle. Panoramic ultrasound images were obtained by moving the probe steadily and slowly on this transverse plane. The probe remained perpendicular to the skin during ultrasonography. Both gastrocnemius heads were measured and were summed for further analysis. Ultrasound images were analyzed with an image analysis software using a polygon selection tool to outline the muscles manually (Figure 4; Motic Images Plus 2.0, Motic, Hong Kong, China). ICC for fascicle length was 0.971 (95% CI: 0.902–0.991, CV = 12%, SEM = 2.1%), for pennation angle was 0.897 (95% CI: 0.684–0.969, CV = 13%, SEM = 4.1%) and for thickness was 0.868 (95% CI: 0.605–0.960, CV = 11%, SEM = 3.9%). In addition, ICC for gastrocnemius heads CSA was 0.922 (95% CI: 0.752–0.977, CV = 15%, SEM = 4.2%).

#### Range of Motion Assessments

Ankle joint angle was measured at rest and at maximum dorsiflexion, using high resolution photographs taken using a digital camera (Casio EXF1, Casio Computer Co. Ltd., Shibuya, Japan). Anatomical markers were placed on the following points to define the ankle angle: (1) knee: femur-tibia joint line (2) ankle: the most prominent point of lateral malleolus and (3) foot: head of the fifth metatarsal. Ankle angle was determined as the angle created by the intersection of a line joining the knee and ankle markers and a line crossing the heel and the fifth metatarsal. Ankle joint angle at rest was measured with the athletes lying in prone position on a physiotherapy bed with the foot hanging loosely off the bed. To minimize parallax error, the leg measured was positioned on a line drawn on the bed, that was parallel to the camera’s field of view (optical axis at 90° with the long axis of the bed). Also, the height of the camera was adjusted so that it was at the level of the bed, and the optical axis of the camera was pointing to mid-calf length. The distance of the camera from the markers was 1.2 m, so that the photograph contained only the lower limb (knee joint, shank and foot).

Measurements of ankle angles during stretching (maximum dorsiflexion) were performed with the athletes standing, facing a wall, with their hands placed against it (Jung et al., 2009). No ankle pre-conditioning movements were performed. The foot was placed on a line drawn on the floor, so that the line passed from the center of the heel and the mid toe. This line was parallel to the field of view of the camera. The position of the body was standardized during the preliminary visits, so that the ankle, knee, hip and shoulder joints were aligned, and the arms were parallel to the ground, while the distance of the foot from the wall was increased until the heel was still in contact with the ground and aligned with the line on the ground. Participants achieved a stretch intensity to the point of discomfort, 8–9 on the scale, using a visual 0–10 Wong Baker FACES Pain Scale that is commonly used in pediatric populations (Wong and Baker, 1988). This stretching position was kept for 1 min. The front leg served only for balance and support. Camera was placed at a height of 20 cm (at around mid-calf length, its horizontal position was such that the optical axis coincided with the mid-calf length). The set-up protocol (leg, joint and camera positions) was standardized for every individual and was kept the same for all images taken throughout the study. All images were analyzed *via* analysis software (Tracker 4.91© 2020 Douglas Brown). ICC for ROM was 0.962 (95% CI: 0.874–0.989, CV = 5%, SEM = 1.1%).

#### Static Stretching Training

**Figure 5:**
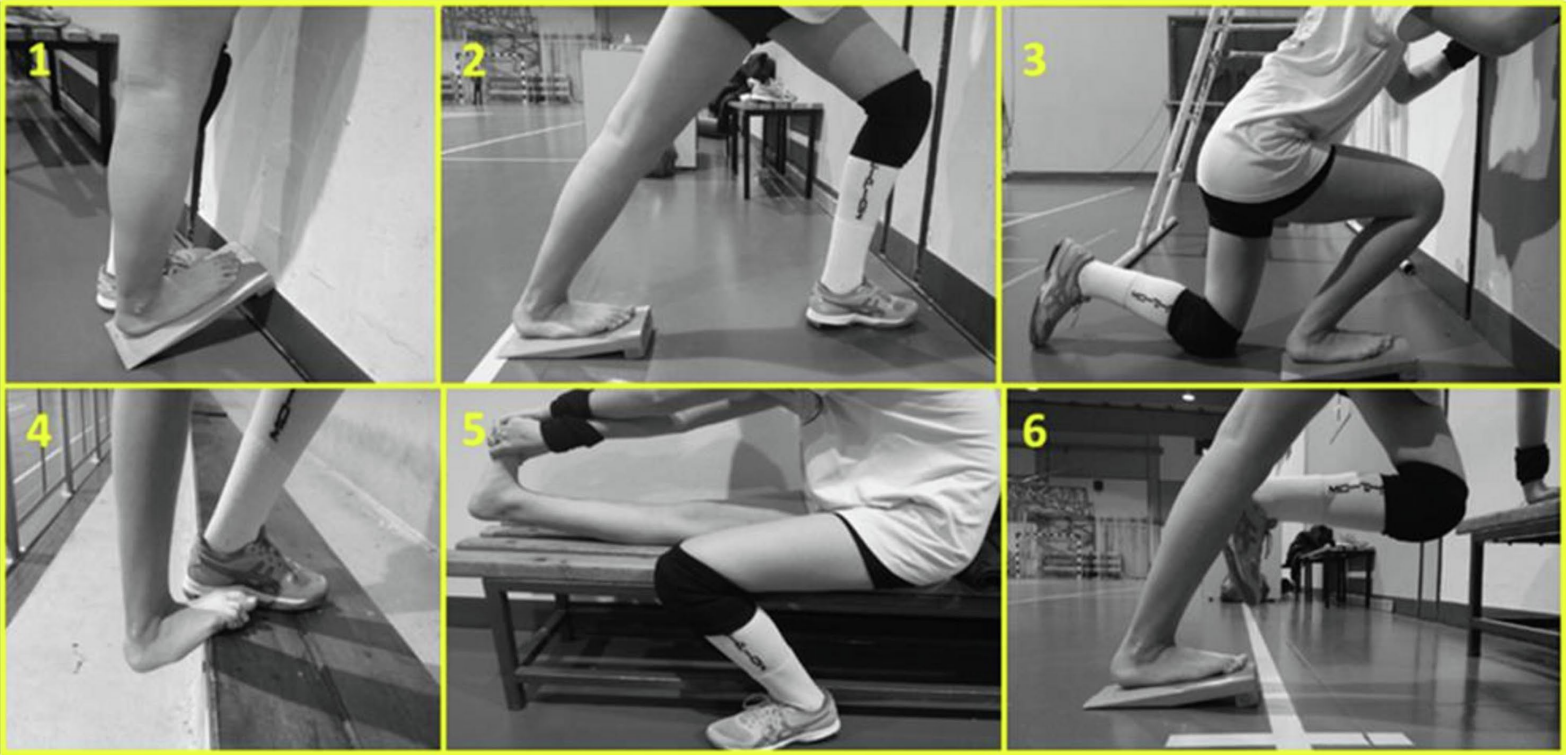
Static stretching exercises targeting mainly triceps surae (1, 2, 4, 6), soleus (3) and hamstrings and triceps surae (5, 6).

The static stretching intervention included five sessions per week, over a 12-week period (60 sessions) and was held under the constant supervision of two experienced coaches and members of the research group. Four times per week participants performed the stretching training in addition to their volleyball training and one more time, they visited the training facilities to perform only stretching training. Each stretching training session consisted of two sets of six static stretching exercises performed using a stretching board and without intervals before volleyball training (Figure 5). During the first 3 weeks, each stretching session was performed for two sets of six repetitions, with each repetition lasting 45 s. The duration of each stretching bout was extended by 15 s every 3 weeks, except for the last 3-weeks (60, 75, 90 and 90 s of stretching bout, for weeks 3, 6, 9, and 12, respectively). Total stretching duration per session was 540 s, 720 s, 900 s at weeks 0–3, 3–6, 6–12, respectively, resulting in a total stretch time of 2,700 s per week for the initial 3 weeks, which gradually increased to 4,500 s per week during the last 6 weeks. During the last 3 weeks athletes maintained stretching intensity by moving their body mass forward. Athletes achieved in each stretching bout an intensity near the point of discomfort, 8–9 by using the visual scale 0–10 of Wong Baker FACES Pain Scale, as described above (Wong and Baker, 1988). The control leg did not perform any type of stretching and was only submitted to typical volleyball training. The stretching protocol was designed to target all the tissues involved in gastrocnemius stretching (e.g., soleus and gastrocnemius deep fasciae, sciatic nerve), because non-muscular structures, such as nervous tissue and fasciae are important for the limitation of joints’ maximal ROM and for pain perception (Nordez et al., 2017; Figure 5).

#### Statistical Analysis

SPSS statistical software (IBM SPSS Statistics Version 22.0, IBM Corporation, Armonk, NY, United States) was used to conduct statistical analyses. Descriptive statistics were calculated for each tested variable. Shapiro Wilk’s test confirmed the normal distribution of the data and paired *t*-test examined pre-intervention differences between the stretched and the control legs in fascicle length at the middle and the distal parts of gastrocnemius medialis and lateralis, ankle angle and jump heights to randomize athletes’ legs. Paired *t*-test also examined differences in anthropometric and maturity characteristics of the athletes pre- and post-intervention.

Three-way ANOVA examined changes pre- and post-intervention and during detraining in ankle angle. Similarly, three-way ANOVA (time × condition × leg) with repeated measures for time (0, 12, 15 weeks), leg (STR or CON) and condition (at rest or at maximum dorsiflexion) was conducted separately for the MP and the DP of gastrocnemius medialis and lateralis to examine changes in fascicle length, pennation angle and thickness. Changes in CMJ height pre- and post-intervention were examined with two-way ANOVA (time × leg) with repeated measures for time (0 and 12) and leg (STR or CON). Similarly, two-way ANOVA (time × leg) with repeated measures for time (0, 12, 15) and leg (STR or CON) examined changes in CSA. When a significant main effect or interaction was found, Tukey *post-hoc* tests were conducted (*p* < 0.05). For pairwise comparisons, effect sizes were determined by Cohen’s *d* (trivial: < 0.19), small (0.20–0.49), medium (0.50–0.79), and large (>0.80; Cohen, 1992). ICCs (intra-day) were used to assess test–retest reliability. Additionally, the standard error of measurement (SEM) and the coefficient of variance (CV) were calculated. Statistical significance was set at *p* < 0.05.

## Results

### Baseline Values

As shown in Table 2, no difference was found between the STR and the CON in all the examined variables (*p* > 0.184).

### Ankle Joint Angles at Rest and During Static Stretching

A main effect for time (*p* < 0.001, *η*^2^ = 0.523), condition (*p* < 0.001, *η*^2^ = 0.993) and leg (*p* < 0.001, *η*^2^ = 0.631) was found for ankle angle. Additionally, leg × time (*p* < 0.001, *η*^2^ = 0.578), time × condition (*p* < 0.001, *η*^2^ = 0.867) and condition × leg (*p* < 0.001, *η*^2^ = 0.749) interactions were observed. Finally, a leg × condition × time interaction was found (*p* < 0.001, *η*^2^ = 0.513; Table 3). *Post-hoc* comparisons showed that resting ankle angle increased from pre- to post-intervention (*p* < 0.001) in both legs, with no difference between legs (*p* = 1.000), and this improvement was maintained during detraining (*p* = 1.000). Ankle dorsiflexion increased in both legs from pre- to-post intervention (*p* < 0.001), with a greater increase observed in the STR than CON (22 ± 20 vs. 8 ± 17%, respectively, *p* < 0.001, 95%CI: 13.4–30.6% vs. 0.7–15.3%). Increases in ankle angles at rest and at maximal dorsiflexion were maintained during detraining (*p* > 0.821), as well as the difference between legs at maximum dorsiflexion (*p* < 0.001; Table 3).

**Table 3:** Ankle angle at rest and during stretching at week-0, week-12 and week-15.

	Leg	Week-0 (^0^)	Week-12 (^0^)	Week-15 (^0^)	*p*	Cohens’ *d* (weeks)		Cohens’ *d* (between legs)		
						0–12	12–15	0	12	15
Rest	CON	113.3 ± 6.2	116.8 ± 5.5^§^	117.1 ± 5.6^†^	<**0.001**	−0.61	−0.18	0.03	0.03	0.18
	STR	113.2 ± 5.9	116.7 ± 6.1^§^	116.8 ± 5.6^†^		−0.60	−0.02			
Stretch	CON	62.8 ± 3.7	57.6 ± 3.0^§^	56.6 ± 3.9^†^		1.58	0.35	0.00	2.43	1.82
	STR	62.8 ± 3.4	49.9 ± 4.0^§^^,^^*^	50.4 ± 3.7^†^^,^^*^		3.77	−0.32			

* *p* < 0.01 from the corresponding control leg value;

§ *p* < 0.05 from the corresponding pre-intervention value to post-intervention value;

† *p* < 0.05 from the corresponding post-intervention value until the end of the detraining period;

CON, Control Leg; STR, Stretched Leg.

### Muscle Fascicle Length at Rest and During Stretching (Gastrocnemius Medialis)

**Figure 6:**
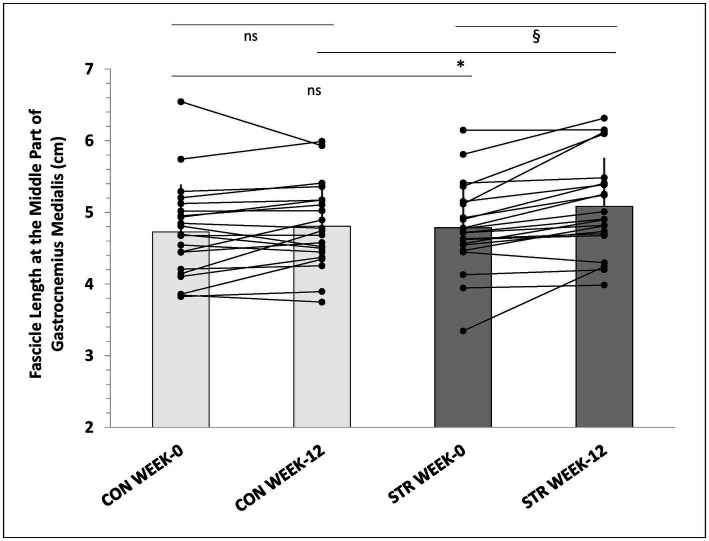
Individualized values of fascicle length in the medial part of gastrocnemius medialis, at week-0 and week-12. *: *p* < 0.01 from the corresponding control leg value; §: *p* < 0.05 from the corresponding pre-intervention value to post-intervention value; CON, control leg; STR, stretched leg.

In the MP of gastrocnemius medialis main effects for time (*p* < 0.001, *η*^2^ = 0.394), condition (*p* < 0.001, *η*^2^ = 0.976) and leg (*p* = 0.007, *η*^2^ = 0.311) were observed (Table 4). Furthermore, leg × time (*p* = 0.002, *η*^2^ = 0.268), time × condition (*p* = 0.014, *η*^2^ = 0.192), and leg × condition × time interactions were found (*p* = 0.021, *η*^2^ = 0.176). *Post-hoc* comparisons (Tukey HSD) showed that resting fascicle length in the MP of the STR increased from pre- to post-intervention (*p* = 0.006) compared to CON (6 ± 7% vs. 2 ± 6%, *p* = 0.014, 95%CI: 3.0–9.0% vs. -0.5-4.5%), and this increase was maintained during detraining (Table 4). Similarly, greater fascicle length at maximum dorsiflexion was observed in the MP of the STR compared to CON (11 ± 7% vs. 3 ± 8% *p* < 0.001, 95%CI: 8.0–14.0% vs. -0.2–6.2%) and this increase was maintained during detraining (*p* = 0.241). In addition, individualized values of MP of gastrocnemius medialis at rest (week-0 and week-12) are shown in Figure 6.

**Table 4:** Gastrocnemius medialis and lateralis fascicle length at rest and during stretching in the middle and the distal part of the muscles, at week-0, week-12 and week-15.

			Leg	Week-0 (cm)	Week-12 (cm)	Week-15(cm)	*p*	Cohens’ *d* (weeks)		Cohens’ *d* (between legs)		
								0–12	12–15	0	12	15
Medialis	Middle Part	Rest	CON	4.7 ± 0.7	4.8 ± 0.6	4.8 ± 0.6	**0.021**	0.13	−0.07	0.08	0.44	0.20
			STR	4.8 ± 0.6	5.1 ± 0.7^§^^,^^*^	4.9 ± 0.6^†^		0.48	−0.33			
		Stretch	CON	6.5 ± 0.9	6.7 ± 0.9	6.6 ± 0.7		0.22	−0.19	−0.04	0.51	0.33
			STR	6.5 ± 0.8	7.2 ± 1.0^§^^,^^*^	7.0 ± 0.8^†^^,^^*^		0.77	−0.44			
	Distal Part	Rest	CON	4.5 ± 0.7	4.6 ± 0.7	4.7 ± 0.6	0.250	0.12	0.16	0.05	0.50	0.22
			STR	4.5 ± 0.6	4.9 ± 0.7	4.8 ± 0.6		0.62	−0.16			
		Stretch	CON	6.2 ± 0.9	6.5 ± 0.8	6.4 ± 0.7		0.34	−0.07	−0.04	0.37	0.55
			STR	6.2 ± 0.9	6.8 ± 0.9	6.8 ± 0.8		0.73	0.06			
Lateralis	Middle Part	Rest	CON	4.7 ± 0.6	4.9 ± 0.6	4.9 ± 0.7	0.484	−0.17	0.00	−0.24	0.56	0.42
			STR	4.8 ± 0.8	5.3 ± 0.7	5.2 ± 0.7		−0.62	0.16			
		Stretch	CON	5.9 ± 0.9	6.2 ± 0.9	6.4 ± 0.9		0.36	0.25	0.56	0.86	0.54
			STR	6.3 ± 0.8	7.0 ± 1.0	6.9 ± 1.0		0.75	−0.09			
	Distal Part	Rest	CON	4.4 ± 0.6	4.6 ± 0.7	4.6 ± 0.7	**0.036**	0.38	0.03	0.28	0.40	0.30
			STR	4.6 ± 0.8	4.9 ± 0.7	4.9 ± 0.8		0.45	−0.03			
		Stretch	CON	5.8 ± 0.8	6.1 ± 0.9	6.3 ± 0.9		0.36	0.20	0.23	0.79	0.55
			STR	6.0 ± 0.8	6.9 ± 1.2^§^^,^^*^	6.8 ± 1.0^†^^,^^*^		0.89	−0.12			

* *p* < 0.01 from the corresponding control leg value;

§ *p* < 0.05 from the corresponding pre-intervention value to post-intervention value;

† *p* < 0.05 from the corresponding post-intervention value until the end of the detraining period;

CON, Control Leg; STR, Stretched Leg.

In the DP of gastrocnemius medialis main effects for time (*p* < 0.001, *η*^2^ = 0.496), condition (*p* < 0.001, *η*^2^ = 0.972) and leg (*p* = 0.04, *η*^2^ = 0.189) were observed (Table 4). Furthermore, a leg × time interaction (*p* = 0.02, *η*^2^ = 0.180) was found. No leg × condition × time interaction was found (*p* = 0.250, *η*^2^ = 0.067).

### Muscle Fascicle Length at Rest and During Stretching (Gastrocnemius Lateralis)

In the DP of gastrocnemius lateralis main effects for time (*p* < 0.001, *η*^2^ = 0.602), condition (*p* < 0.001, *η*^2^ = 0.936) and leg (*p* = 0.001, *η*^2^ = 0.460) were found. Additionally, leg × time (*p* = 0.036, *η*^2^ = 0.154), leg × condition (*p* = 0.026, *η*^2^ = 0.225), time × condition (*p* = 0.010, *η*^2^ = 0.208), and leg × condition × time (*p* = 0.036, *η*^2^ = 0.153) interactions were found. *Post-hoc* comparisons (Tukey HSD) showed that fascicle length at maximum dorsiflexion was greater in the DP of the STR compared to the CON at week-12 (15 ± 13% vs. 6 ± 8%, *p* < 0.001, 95%CI: 9.4–20.6% vs. 2.6–9.4%) and this increase was maintained during detraining (*p* = 0.241; Table 4).

### Pennation Angle and Muscle Thickness

#### Gastrocnemius Medialis

Analysis for pennation angle demonstrated a main effect for condition (rest-stretch) in the MP and the DP of gastrocnemius medialis (*p* < 0.001, *η*^2^ = 0.950 and *p* < 0.001, *η*^2^ = 0.921, respectively) but no interaction. Analysis for muscle thickness showed a main time (*p* = 0.002, *η*^2^ = 0.264), leg (*p* = 0.020, *η*^2^ = 0.244) and condition effect (*p* < 0.001, *η*^2^ = 0.905) for the MP and a main time (*p* < 0.001, *η*^2^ = 0.374) and condition effect (*p* < 0.001, *η*^2^ = 0.943) for the DP of gastrocnemius medialis.

#### Gastrocnemius Lateralis

Analysis for pennation angle showed a main time and condition effect for the MP (*p* = 0.003, *η*^2^ = 0.257 and *p* < 0.001, *η*^2^ = 0.779, respectively) and the DP (*p* = 0.001, *η*^2^ = 0.289 and *p* < 0.001, *η*^2^ = 0.858, respectively) of the muscle but no significant interaction. Similarly, analysis for muscle thickness showed a main condition effect for the MP and the DP (*p <* 0.002, *η*^2^ = 0.384 and *p* < 0.001, *η*^2^ = 0.800) but no interaction.

### Gastrocnemius Medialis and Lateralis Anatomical Cross-Sectional Area

**Figure 7:**
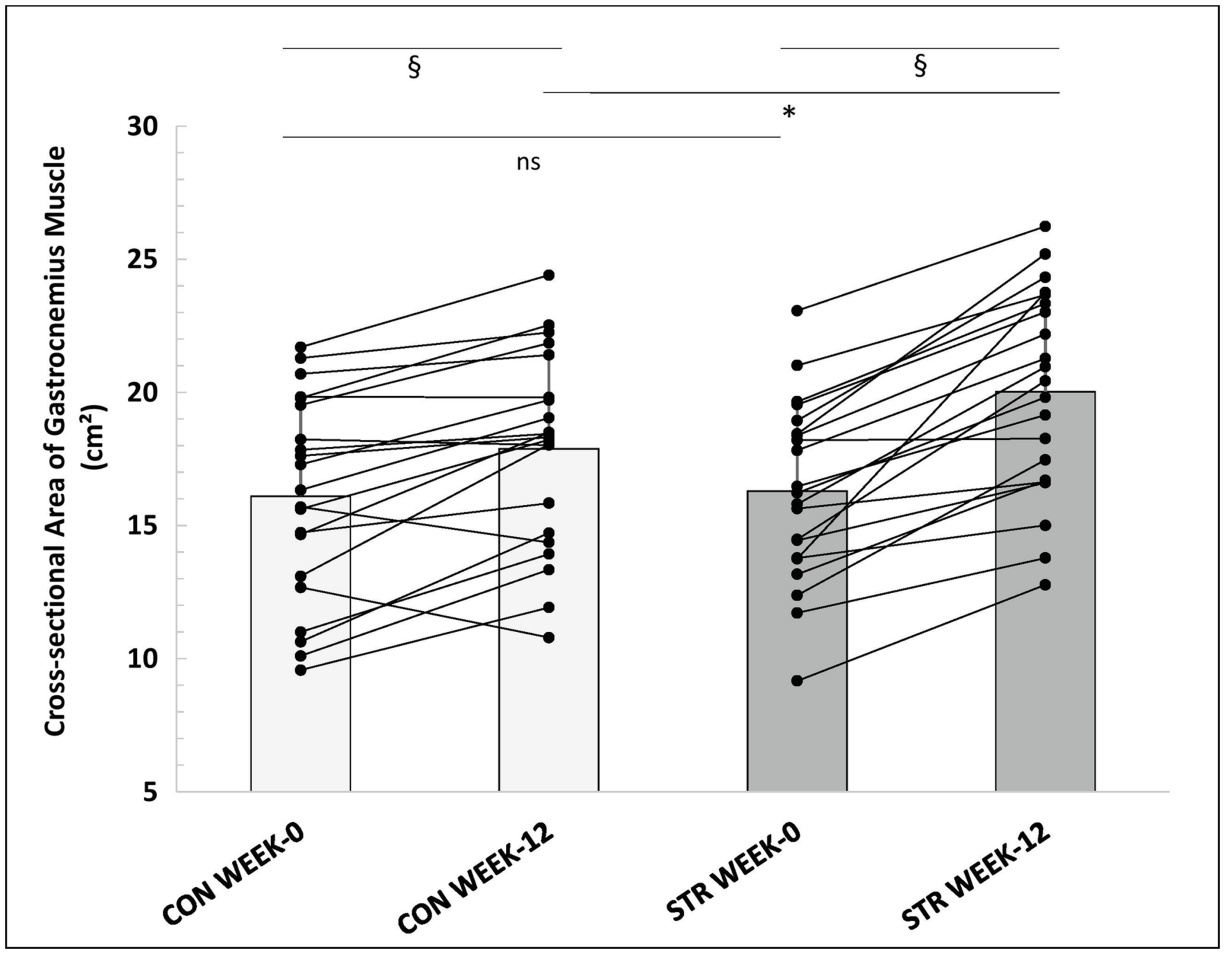
Individualized values of gastrocnemius muscle anatomical CSA (sum of gastrocnemius medialis and lateralis) of the stretched and control legs, at week-0 and week-12. *: *p* < 0.01 from the corresponding control leg value; §: p < 0.05 from the corresponding pre-intervention value to post-intervention value; CON, control leg; STR, stretched leg.

Two-way ANOVA with repeated measures for time (0, 12, and 15 weeks) and leg (STR and CON) examined changes in CSA. A main effect for time (*p* < 0.001, *η*^2^ = 0.663), and leg (*p* < 0.001, *η*^2^ = 0.550) was found. Additionally, a leg × time interaction was observed (*p* < 0.001, *η*^2^ = 0.429). *Post-hoc* comparisons (Tukey HSD) showed that post-intervention CSA increased in both legs (*p* < 0.001), with a greater increase observed in the STR than the CON (23 ± 14 vs. 13 ± 14%, respectively, *p* < 0.001, 95%CI: 17.0–29.0% vs. 7.0–19.0%; Figure 7). Increases in CSA as well as differences between legs were maintained during detraining (*p* > 0.612). Pre- and post-intervention individualized values of gastrocnemius CSA are represented in Figure 7. Effect sizes for the STR and the CON were *d* = 1.05 and *d* = 0.49, respectively, from pre- to post-intervention and *d* = 0.05 and *d* = 0.12, from post-intervention into detraining. In addition, effect sizes for between legs comparisons were *d* = 0.06, *d* = 0.58 and *d* = 0.54, at baseline, week-12 and week-15, respectively.

### One-Leg Countermovement Jump Performance

Two-way ANOVA with repeated measures for time (week 0 and 12) and leg (STR and CON) examined changes in CMJ height. Analysis showed a main effect for time (*p* = 0.002, *η*^2^ = 0.388) and leg (*p* = 0.001, *η*^2^ = 0.425), and a time × leg interaction (*p* = 0.019, *η*^2^ = 0.245). *Post-hoc* comparisons (Tukey HSD) showed that CMJ height improved in both legs post-intervention (from CON: 6.3 ± 1.77 cm to 7.1 ± 1.7, *p* < 0.005 and STR: 6.5 ± 1.8 to 8.1 ± 2.3 cm, *p* < 0.001) however, the increase in the STR was greater than CON (27 ± 30 vs. 17 ± 23% respectively, *p* = 0.001, 95%CI: 14.2–39.8% vs. 7.2–26.8%). Effect sizes for the STR and the CON were *d* = 0.75 and *d* = 0.46, respectively. In addition, effect sizes for between legs comparisons were *d* = 0.49 and *d* = 0.66, at baseline and week 12, respectively. Due to COVID-19 restrictions, no evaluation of CMJ in week 15 was performed.

## Discussion

The main finding of this study was that high-volume unilateral stretching for 12 weeks was effective in inducing greater increases in ankle dorsiflexion, muscle CSA and jumping height than volleyball training alone. Moreover, muscle head specific changes in fascicle length were observed in the STR. Specifically, significant increases were observed in fascicle length at rest and at maximum dorsiflexion in the MP of gastrocnemius medialis. An increase in fascicle length at maximum dorsiflexion was also found in the DP of gastrocnemius lateralis. Notably, changes in ankle dorsiflexion, fascicle length and CSA were maintained for 3 weeks after the end of stretching training.

### Ankle Angle at Maximum Dorsiflexion

Ankle dorsiflexion angle increased in both legs with a larger increase observed in the STR than the CON (22 vs. 8%, respectively). The improvement of ankle dorsiflexion in the STR from pre- to post-intervention agrees with recent evidence in adults (an increase of 24%; Longo et al., 2021). Interestingly, a bilateral increase in ROM after a 6-month unilateral stretching has been recently reported in the study of Moltubakk et al. (2021). Some previous studies demonstrated bilateral gains following an acute unilateral stretching exercise (Marchetti et al., 2014) while long-term observations found that ROM was unchanged in the control leg (Akagi and Takahashi, 2014). However, previous studies lasted from 3 to 9 weeks or used short-duration protocols (e.g., 90 s of stretching, three times per week; Donti et al., 2021) while in the present study, increases in ankle dorsiflexion were observed following 12-weeks of high-volume stretching. Behm et al. (2021) reported that stretch durations longer than 240 s demonstrated larger non-local ROM increases than lower durations, in acute stretching interventions. As the changes in the contralateral leg found in this study were observed in the absence of morphological changes, the bilateral response to stretching may be attributed to central neural adaptations (i.e., increased pain tolerance; Trajano et al., 2017), similar to cross-education effects reported in strength training studies (Manca et al., 2017). It may also be speculated that, volleyball training for 12 weeks enhanced ankle dorsiflexion overtime in the CON leg although, lower values of ankle dorsiflexion were reported in adult compared to child female volleyball players in previous cross-sectional studies (Donti et al., 2019; Panidi et al., 2020).

Increases in ankle dorsiflexion were maintained during detraining, in both legs. The retention of joint ROM for a given period is important, however, previous evidence in youth athletes is limited. It is reported that levels of flexibility tend to plateau or even decrease at the time of the adolescent growth spurt (Malina, 2007), indicating that the maintenance of previously acquired joint ROM may be the training focus for future athletic development. Furthermore, at week 15, ankle dorsiflexion angle was larger in the STR than the CON, suggesting that except neural, structural changes may also be associated with increased joint ROM. A potential association between ROM and fascicle length is only reported when considering muscle slack angle between individuals (Hirata et al., 2021), therefore, the relative contribution of fascicle length to joint ROM enhancement and retention cannot be explained with the present study design.

### Ankle Angle at Rest

At rest, ankle angles were similar between the STR and the CON leg. However, post-intervention an increase in resting ankle angle of both legs was observed, which was maintained during detraining. Resting angle joint is determined by the sum of all the torques acting around the joint (Moltubakk, 2019). In a cross-sectional study comparing triceps surae architectural properties in ballet dancers and controls, Moltubakk et al. (2018) found similar resting ankle joint ROM between the two groups and assumed that the different operating fascicle length of the dancers depended on tissue material properties and did not imply different slack length of the tissues surrounding the joint. However, whether, high-intensity, long-term stretching interventions imply alterations in tissue slack length, and changes in the ‘neutral’, resting ankle joint angle, is not known. It is plausible that ‘neutral’ ankle joint ROM is affected by chronic stretching, a finding typically observed in dancers and gymnasts (Steinberg et al., 2006).

### Muscle Architectural Adaptations

An important finding of the present study was the significant increase in resting fascicle length in the MP of gastrocnemius medialis, by 6%, while no increase was found in fascicle length in gastrocnemius lateralis. Similarly, Andrade et al. (2020) reported an increase in resting fascicle length of gastrocnemius medialis by 0.4 cm (*p* = 0.017) following 12 weeks of stretching. In that study, a tendency for an increase was found in gastrocnemius lateralis fascicle length, albeit non-significant (Andrade et al., 2020). Fascicle length is thought to reflect the number of in series sarcomeres (Lieber and Fridén, 2000) and is related to maximal muscle excursion and the muscle length at which the myofilaments optimally overlap (Gordon et al., 1966). Long-term immobilization in lengthened position in animals resulted in an increase in muscle size and fascicle length through the addition of sarcomeres in series (Tabary et al., 1972). Changes in sarcomere numbers in animals were dependent upon the muscle length and duration of the immobilization (Tabary et al., 1972). However, an increase in fascicle length following stretching in humans has not been clearly demonstrated up to now. Blazevich et al. (2014) did not detect any changes in plantar flexors fascicle length, and tendon elongation, following 3 weeks of static stretching training. Similarly, Nakamura et al. (2021) reported that 5-weeks of stretching did not induce changes in muscle architecture. However, studies that applied more than 8 weeks of stretching and/or a long duration and intense stretching protocol are limited. Simpson et al. (2017) found that 6 weeks of overloaded static stretching (stretching in a leg press machine loaded to 20% of the subjects’ maximal voluntary contraction) increased medial and lateral gastrocnemius fascicle length and thickness. Freitas and Mil-Homens (2015) also found a significant increase in biceps femoris fascicle length in physically active participants, following 8 weeks of intensive static stretching training (450 s of stretching repeated three times per week). Along this line, the duration that the muscle was held under stretch and at a long length in this study (540 s increasing to 900 s) was much longer than in previous studies. Resistance training studies indicated that increasing the time of muscle under tension induces muscle hypertrophy and enhances muscle strength as a response to high mechanical stress on tendons and muscles (Toigo and Boutellier, 2006). It may be postulated that even the low levels of muscle strain, induced by long duration and high-volume static stretching may trigger mechanotransduction signaling pathways associated with changes in muscle architecture (Mohamad et al., 2011). This result is interesting because previous studies in growing children reported that medial gastrocnemius muscle growth in adolescence is mediated by increased fascicle diameter rather than longitudinal fascicle growth (Weide et al., 2015) due to the increased serum levels of growth factors (Binzoni et al., 2001). It is reported that fascicle length increases by 1% per year, in typically developing children aged 5–12 years old (Bénard et al., 2011) although evidence on fascicular length changes during growth is limited. In this study, resting fascicle length in the MP of gastrocnemius medialis increased by 6%. Thus, it may be speculated that long-term, high-volume stretching training can modify the growth pattern of muscle geometry in developing athletes, through increases in fascicle length and extensibility. Notably, the unilateral stretching intervention used in this study for 12 weeks, included exercises that also targeted non-muscular structures (e.g., fascia, and peripheral nerves) which are suggested to limit the maximal ROM in some multi joint positions (Nordez et al., 2017).

Previous cross-sectional studies observed greater fascicle elongation in flexible compared to inflexible subjects (Blazevich et al., 2012; Moltubakk et al., 2018) and stretching interventions reported increased muscle extensibility after 3 or 4 weeks of static stretching training (Nakamura et al., 2012; Blazevich et al., 2014). The greater fascicle length at maximum dorsiflexion that was observed in the STR compared to the CON in the MP of gastrocnemius medialis and the DP of gastrocnemius lateralis confirm previous evidence suggesting increased muscle extensibility after 3 or 4 weeks of static stretching training (Nakamura et al., 2012; Blazevich et al., 2014). An increase in gastrocnemius medialis muscle elongation (by 42%), accompanied by an increase in ROM was also reported recently in the study of Longo et al. (2021). Increased fascicle extensibility may also be attributed to changes in properties of intramuscular connective tissues (e.g., perimysium) that are related to a decrease in passive stiffness (Purslow, 1989).

Fascicle length changes in this study occurred in the MP of gastrocnemius medialis and in the DP of gastrocnemius lateralis. Gastrocnemius lateralis has an oblique attachment to the Achilles tendon while gastrocnemius medialis is linear and experiences greater relative loads during movement. Due to the oblique attachment of gastrocnemius lateralis, a portion of the tension is dissipating along the angle of attachment (Simpson et al., 2017). Therefore, the different adaptations to fascicle stretch between gastrocnemius medialis and lateralis found in this study, may be due to the unequal loading of the two heads, a result also reported in previous research (Simpson et al., 2017).

No changes were found in this study in gastrocnemius medialis pennation angles in both legs, while in gastrocnemius lateralis, pennation angles decreased. Simpson et al. (2017) also reported unchanged pennation angles in gastrocnemius medialis and decreased pennation angles in gastrocnemius lateralis following 6 weeks of overloaded static stretching training and assumed that decreases in pennation angle in gastrocnemius lateralis contributed to torque maintenance due to the preferential passive loading of gastrocnemius lateralis when all plantar flexors are involved (Arndt et al., 1999). In contrast, in the present study, gastrocnemius medialis muscle thickness in the MP and the DP increased in both legs, a finding possibly associated with a concomitant increase in CSA due to growth and volleyball training. Similarly, Moltubakk et al. (2021), reported a bilateral increase in gastrocnemius medialis thickness despite the unilateral stretching intervention used in the study.

### Anatomical Cross-Sectional Area

Following intervention, CSA increased in both legs with a larger increase observed in the STR than CON (by 23 and 13% respectively). To the authors’ knowledge, no previous study examined the effect of stretching training on gastrocnemius muscle anatomical CSA in humans. CSA reflects the size of a muscle, estimates muscle hypertrophy or atrophy and is a determinant of force production (Franchi et al., 2018). Gastrocnemius muscle geometry adapts to growth predominantly by increasing the length component of the physiological CSA with smaller increases in fascicle length (Weide et al., 2015). Although, maturational changes during the 3-months intervention and supplementary volleyball training may also have affected increases in CSA, the net difference between legs can be attributed to muscle morphological changes following stretching training. In addition to the time of a muscle being held under stretching, static stretching causes ischemia (McCully, 2010) (attributed to the local pinching of blood vessels), which in turn, induces changes in metabolic sensors that regulate muscle growth (McCully, 2010). Interestingly, ankle dorsiflexion increases in this study were proportional to CSA increases (22 and 23%, for the STR vs. 8 and 13%, for the CON, respectively) indicating that the ankle operating ROM may be related to muscle morphological adaptations to functional demands.

Changes in fascicle length at rest and at maximum dorsiflexion in the MP of gastrocnemius medialis and the DP of gastrocnemius lateralis and in CSA were maintained for 3 weeks into detraining but increases were more pronounced in the STR than CON. A recent study reported that the training effects of stretching training in healthy males enhanced joint ROM and reduced muscle stiffness, but changes were dismissed after an equal period of detraining (Nakamura et al., 2021). However, in that study stretching training lasted for 5 weeks and was followed by 5 weeks of detraining while in our study 12 weeks of high-volume stretching training were performed and were followed by 3 weeks of detraining. In addition, the participants of this study continued their volleyball training thus, athletes applied strength throughout the acquired ROM, and this may have contributed to the retainment of adaptations in that period.

### Jumping Height

Previous research on the effects of long-term stretching training on measures of force and power generation reported conflicting results. For example, Moltubakk et al. (2021) found decreased passive torque and joint stiffness at comparable dorsiflexion angles, after 26 weeks of stretching training in healthy females. In contrast, Longo et al. (2021) reported that 12 weeks of stretching intervention did not alter maximum voluntary contraction and rate of torque development in the first 200 ms of muscle contraction, in nine males and six females. In the present study, one-leg CMJ height increased post-intervention in the STR and CON with a larger increase observed in the STR than CON (27 vs. 17%, respectively). Bilateral jump height increases may be attributed to maturational changes following the 12-weeks period, as also reported in previous studies (Moeskops et al., 2020) and to the systematic additional volleyball training. Although no association was found in this study between CMJ performance and changes in ROM, it is suggested that improvements in ankle dorsiflexion may allow for a better production, storage, and redistribution of elastic strain energy (Kubo et al., 2001) and a recent cross-sectional study in adults reported medium correlations between jump height and ankle dorsiflexion (Konrad et al., 2021). In addition, the changes in resting fascicle length and muscle CSA found in this study, may indicate changes in muscle morphology following stretching training. The combination of increased dorsiflexion with gastrocnemii structural adaptations may modify contractile function and enhance muscle power production as gastrocnemius is an important power generator for jumping movements (Earp et al., 2010). In line with this result, Kokkonen et al. (2007) also reported improved jump performance and Yahata et al. (2021), positive changes in muscular strength following stretching training.

## Limitations

This study has some limitations that should be acknowledged. First, the participants of this study were female adolescent volleyball athletes, so the findings cannot be generalized to other age groups with different physical activity levels. In addition, the participants were growing girls, and therefore increases in hormonal levels affecting muscle mass and collagen metabolism should be further examined. External factors, such as maturation changes and supplementary volleyball training may have also influenced the current results, but these factors would affect both legs similarly. Another limitation is that muscle electromyographical activity, passive force and other neuromuscular characteristics were not measured, and this prevents further interpretation of the observed increase in CMJ. In addition, fascicle length was measured at maximum dorsiflexion and not at standardized angles, thus, differences in muscle extensibility may also be related to changes in ankle ROM (Moltubakk et al., 2021). Cross-over effects may also influence bilateral changes, however, the unilateral design used in this study, allowed us to conclude that our results were due to intervention rather than systematic error.

## Conclusion

Our findings showed that, in female adolescent athletes, high-volume unilateral stretching for 12 weeks increases ankle dorsiflexion, fascicle length and muscle CSA more than volleyball training alone, and these adaptations may partly explain improvements in jump performance. Stretching training induces heterogeneous changes across muscle length or between muscles. ROM also increased in the control leg, indicating a cross-over effect of stretching. Notably, changes in ankle dorsiflexion, fascicle length and CSA were maintained following detraining.

## Data Availability Statement

The raw data supporting the conclusions of this article will be made available by the authors, without undue reservation.

## Ethics Statement

The studies involving human participants were reviewed and approved by School of Physical Education and Sport Science of National and Kapodistrian University of Athens Bioethics Committee (registration number: 1040, 14/02/2018). Written informed consent to participate in this study was provided by the participants’ legal guardian/next of kin.

## Author Contributions

IP and OD conceived the experiment, interpreted the data, and drafted the manuscript. IP, OD, GB, and AD designed the experiment. IP and VG recruited the subjects. IP, OD, AD, VG, and GT performed the experiments. IP and VG analyzed the data. OD, AK, and GB substantially contributed to data analysis. GB, GT, AK, and AD made important intellectual contributions during revision. All authors approved the final version of the manuscript and agreed to be accountable for the content of the work.

### Conflict of Interest

The authors declare that the research was conducted in the absence of any commercial or financial relationships that could be construed as a potential conflict of interest.
